# Actinidin-Enriched Kiwifruit Concentrate (Kwd+^®^) Improves Protein Utilization Efficiency and Modulates Body Composition and Muscle Function in a Protein Source-Dependent Manner

**DOI:** 10.3390/ijms27167086

**Published:** 2026-08-07

**Authors:** Iván Benito-Vázquez, Pablo Méndez-Albiñana, Aaron Fernández-Quintero, María Ines Morán-Valero, Francisco Javier Moreno, Marina Díez-Municio, Nuria Fernández, Luis Monge, Jose Antonio Uranga-Ocio, Javier Blanco-Rivero

**Affiliations:** 1 Food Science Research Institute CIAL (CSIC-UAM), Nicolás Cabrera 9, 28049 Madrid, Spain; ivan.benito@pharmactive.eu (I.B.-V.); pablo.mendez.a@csic.es (P.M.-A.); javier.moreno@csic.es (F.J.M.); 2 Pharmactive Biotech Products SLU, Faraday, 7, 28049 Madrid, Spain; aaron.fernandez@pharmactive.eu (A.F.-Q.); ines.moran@pharmactive.eu (M.I.M.-V.); mdiez@pharmactive.eu (M.D.-M.); 3 Department of Physiology, School of Medicine, Autonomous University of Madrid (UAM), 28029 Madrid, Spain; nuria.fernandez@uam.es (N.F.); luis.monge@uam.es (L.M.); 4 Department of Basic Health Sciences, University Rey Juan Carlos (URJC), 28922 Alcorcón, Spain; jose.uranga@urjc.es; 5 High Performance Research Group in Physiopathology and Pharmacology of the Digestive System (NeuGut), University Rey Juan Carlos (URJC), 28922 Alcorcón, Spain; 6 Research Institute of La Paz University Hospital (IdiPAZ), 28040 Madrid, Spain; 7 Center for Biomedical Research Network (CIBER) in Cardiovascular Diseases (Ciber-CV), 28029 Madrid, Spain

**Keywords:** gastric digestion, kiwifruit protease, dietary protein quality, amino acid availability, muscle performance, metabolic efficiency

## Abstract

Protein utilization efficiency is a key determinant of metabolic health, body composition, and muscle function, particularly under high-protein dietary conditions and when using plant-based protein sources with lower digestibility. However, the extent to which enhancing early gastric proteolysis translates into improved whole-body protein utilization remains unclear. The present *in vivo* study investigated whether supplementation with an actinidin-enriched kiwifruit concentrate (Kwd+^®^) modulates protein utilization efficiency and physiological outcomes under normoproteic and hyperproteic dietary conditions using casein or pea protein as dietary sources. Rats were fed normoproteic casein (NP), high-protein casein (CHP), or high-protein pea (PHP) diets with or without Kwd+^®^ supplementation for 8 weeks. Gastric protein hydrolysis patterns were evaluated by SDS–PAGE, while metabolic outcomes were assessed through body composition, plasma amino acid profiling, skeletal muscle morphology, and contractile function. Kwd+^®^ supplementation enhanced gastric proteolysis in a protein source-dependent manner. Despite the absence of significant changes in circulating amino acid concentrations after multiple comparison correction, Kwd+^®^ improved markers of protein utilization efficiency depending on dietary protein source. In animals fed a high-protein pea diet, supplementation significantly reduced fat mass relative to accumulated protein intake, indicating improved nutrient partitioning. In contrast, in animals fed a high-protein casein diet, Kwd+^®^ increased muscle mass relative to protein intake, suggesting enhanced anabolic efficiency. Under normoproteic conditions, Kwd+^®^ supplementation was associated with an increased muscle fiber cross-sectional area and improved fatigue resistance without alterations in fiber type composition or contractile protein abundance. These findings demonstrate that modulation of early gastric proteolysis through actinidin produces protein source-dependent effects on protein utilization efficiency, nutrient partitioning, and muscle function. This work highlights a novel nutritional strategy to improve metabolic outcomes and muscle performance, particularly in the context of high-protein and plant-based diets.

## 1. Introduction

Efficient dietary protein digestion is essential for optimal amino acid bioavailability and the stimulation of skeletal muscle protein synthesis (MPS), a process tightly regulated by essential amino acid availability and intracellular signalling pathways [1,2]. Protein digestion is initiated in the stomach, where hydrochloric acid induces protein denaturation and pepsin begins proteolytic cleavage, influencing the subsequent rate of amino acid delivery to the small intestine. The speed of gastric digestion and amino acid absorption significantly modulates postprandial whole-body protein synthesis, breakdown, and net protein balance, as demonstrated by differential kinetic responses to slow and fast proteins [3].

Increased dietary protein intake is frequently recommended in athletic populations aiming to support lean mass accretion and in older adults seeking to attenuate age-related anabolic resistance [4]. However, evidence indicates that increasing protein intake beyond recommended levels does not result in a proportional increase in muscle protein synthesis, suggesting a non-linear relationship between protein dose and anabolic response [5]. Furthermore, protein quality, digestibility, and amino acid composition significantly influence postprandial anabolic potential, as plant-based proteins generally exhibit lower digestibility and essential amino acid availability compared with animal-derived proteins [6]. Importantly, the rate of protein digestion and amino acid absorption modulates the magnitude and timing of postprandial aminoacidemia, thereby influencing systemic amino acid availability [7]. Under aging conditions, skeletal muscle exhibits reduced sensitivity to essential amino acids, a phenomenon termed anabolic resistance, characterized by diminished activation of anabolic signalling pathways and impaired stimulation of MPS [8,9]. This reduced responsiveness may necessitate higher protein intakes to maintain muscle mass and function in older individuals [10].

Collectively, these considerations underscore the importance of optimizing early-phase protein digestion and amino acid delivery kinetics to support efficient muscle protein accretion, particularly under hyperproteic or age-associated conditions in which protein utilization efficiency may become physiologically relevant.

In this context, strategies aimed at enhancing early-phase gastric proteolysis have emerged as a potential approach to improve protein digestion efficiency and amino acid delivery kinetics.

Kiwifruit (*Actinidia* spp.) is distinguished among fruits by the high abundance of its endogenous cysteine protease, actinidin (EC 3.4.22.14), which accounts for approximately 50–60% of the soluble protein fraction in green kiwifruit (*Actinidia deliciosa* cv. Hayward) [11,12]. Proteomic and developmental analyses confirm that actinidin represents the dominant proteolytic activity throughout fruit maturation [13]. Structurally, actinidin belongs to the papain-like (C1) family of cysteine proteases and shares conserved catalytic residues characteristic of this group [14,15]. Biochemical characterization reports a molecular mass of approximately 23–27 kDa and activity over a broad pH range, with optimal activity described under mildly acidic conditions compatible with gastric digestion [11,12,16,17,18]. Comparative studies of plant proteases highlight kiwifruit as one of the richest natural sources of food-grade cysteine proteolytic activity [19]. Functionally, actinidin exhibits broad substrate specificity, effectively hydrolysing animal and plant proteins [20], a property that underpins its proposed role in modulating gastric protein digestion. Beyond its enzymatic activity, kiwifruit composition and protease abundance vary across species and cultivars, although *A. deliciosa* remains the principal dietary source of active actinidin [21].

Within this context, plant-derived proteases have been investigated as potential modulators of dietary protein digestion. Among these, actinidin has received particular attention due to its demonstrated activity under acidic conditions compatible with gastric digestion. In a controlled *in vitro* gastric model simulating pepsin digestion at pH 1.9, a kiwifruit concentrate containing actinidin significantly enhanced the degradation of several food proteins, including caseins, soy protein isolate, gluten, and beef muscle proteins, as evidenced by accelerated disappearance of intact protein bands and altered peptide patterns on SDS-PAGE [22,23,24]. These findings were later supported by *in vivo* data in the growing rat model, where dietary inclusion of Hayward kiwifruit significantly increased gastric degradability of multiple protein sources, including soy protein isolate, beef muscle protein, gelatin, and gluten, compared with controls. SDS-PAGE analyses of stomach digesta confirmed greater disappearance of intact proteins and increased fragmentation during the gastric phase, whereas effects at the ileal level were comparatively modest [25]. In a physiologically relevant porcine model dietary actinidin accelerated gastric emptying and increased the gastric breakdown of beef muscle proteins, particularly higher-molecular-weight fractions, supporting a role for actinidin in modulating the rate of gastric digestion and the delivery of digesta to the small intestine [26]. Complementing these findings, mechanistic work in the same research line links the rate at which digested nitrogen enters the small intestine (a function of gastric proteolysis and gastric emptying) with patterns of amino acid disappearance along intestinal segments, reinforcing the concept that actinidin can influence digestion–absorption kinetics rather than merely increasing the overall extent of protein degradation [27].

In humans, a controlled trial in older adults reported that co-ingesting Hayward green kiwifruit with a beef meal produced a more rapid rise in peripheral essential amino acids (including branched-chain amino acids), consistent with a kinetic shift in digestion/absorption; importantly, this is best interpreted as altered temporal dynamics rather than definitive proof of increased cumulative nitrogen absorption [28]. Collectively, these animal and human data support a mechanistic framework in which actinidin enhances early-phase gastric proteolysis and can accelerate downstream amino acid availability profiles, while highlighting the need for further controlled studies linking these kinetic effects to functional physiological outcomes.

However, important gaps remain regarding the physiological and functional relevance of actinidin-mediated proteolysis. Although enhanced gastric protein degradation has been demonstrated *in vivo* [25], it remains unclear whether enhanced degradation of gastric macropeptides translates into sustained improvements in intestinal amino acid absorption, systemic amino acid availability, and downstream metabolic efficiency under controlled dietary conditions [3,25,26].

The impact of actinidin may be especially context-dependent, as normoproteic and hyperproteic diets impose distinct constraints on gastric processing and amino acid kinetics [2,8]. Under hyperproteic conditions, where increased protein load may impose greater demands on gastric processing and proteolytic efficiency, improved early-phase hydrolysis could enhance temporal protein utilization efficiency and potentially reduce the proportion of undigested protein delivered to the distal intestine [25,26,27,29].

Conversely, under normoproteic intake, the metabolic consequences may differ, particularly when digestive capacity is unlikely to be rate-limiting [30,31]. Moreover, while accelerated amino acid appearance has been documented, the extent to which this kinetic modulation influences muscle-related outcomes, such as protein utilization efficiency, tissue accretion, or functional adaptation, remains insufficiently characterized [28]. Given that skeletal muscle anabolic responses are sensitive not only to amino acid quantity but also to the rate of amino acid delivery, understanding how actinidin affects digestion–absorption–metabolism coupling is essential for establishing its nutritional relevance [3,32,33].

Therefore, the present study aimed to investigate the differential modulation of animal- and plant-based protein digestion by an actinidin-enriched kiwifruit concentrate (Kwd+^®^) under normoproteic and hyperproteic dietary conditions and to determine whether enhanced gastric proteolysis translates into measurable effects on amino acid availability, protein efficiency, and muscle-related physiological outcomes.

## 2. Results

### 2.1. Body Weight Gain and Feed Intake

Body weight gain was similar across all experimental groups throughout the eight-week period (Figure S2A). Despite consuming considerably more total protein (Figure S2B), hyperproteic animals (CHP and PHP) did not accumulate greater body mass than normoproteic controls (NP). Consistently, the ratio of body weight gain to protein intake increased proportionally in all diets (Figure S2C).

Kwd+^®^ supplementation did not significantly modify weight gain in either the NP or CHP diets at any level of protein intake (*p* > 0.05). In the PHP diet, no significant effect was observed at mean or high protein intake levels; however, a significant simple effect of Kwd+^®^ emerged at low protein intake (−1 SD within diet). PHP animals supplemented with Kwd+^®^ gained significantly more weight than unsupplemented controls (EMM = 34.50, SE = 4.86 vs. EMM = 4.72, SE = 10.00; mean difference = 29.78, 95% CI [7.16, 52.40], SE = 10.99, t(25.33) = 2.71, *p* = 0.012, Bonferroni-adjusted *p* = 0.036, d = 1.73, 95% CI [0.42, 3.04]). The difference-in-differences between PHP and NP reached nominal significance (*p* = 0.046); however, this result was no longer significant after Bonferroni correction (Bonferroni-adjusted *p* = 0.092).

### 2.2. Body Composition

Regarding fat deposition, PHP animals supplemented with Kwd+^®^ showed a significantly lower fat mass-to-accumulated-protein ratio than unsupplemented PHP controls, indicating that Kwd+^®^ supplementation attenuated fat deposition relative to protein consumed in the plant-based high-protein group (Table 1, Panel A). The magnitude of this effect was moderate-to-large (d = −0.98). This effect was not observed in NP or CHP animals, and no significant differences-in-differences were detected between diets (Table 1, Panel B).

For skeletal muscle mass, CHP animals supplemented with Kwd+^®^ showed a significantly higher unstimulated triceps mass-to-accumulated-protein ratio compared with unsupplemented CHP controls, indicating greater efficiency of dietary protein incorporation into muscle tissue under casein-based hyperproteic conditions (Table 2, Panel A). This effect was associated with a very large standardized effect size (d = 2.16). No significant effect was observed in NP or PHP groups, and differences-in-differences between diets were not significant (Table 2, Panel B).

### 2.3. Gastric and Intestinal Protein Digestibility

**Figure 1:**
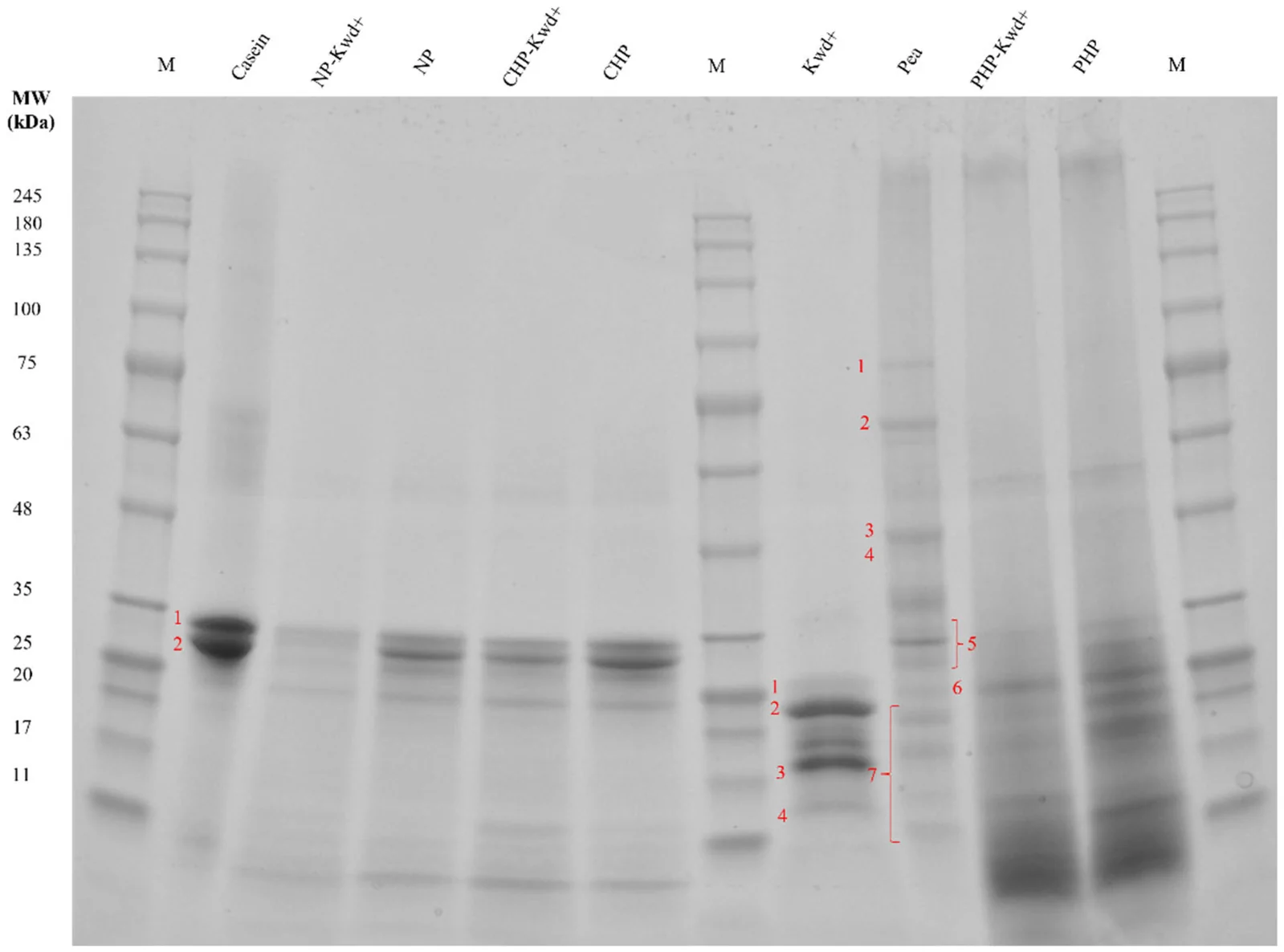
SDS–PAGE profiles of gastric protein hydrolysis patterns in stomach chyme prior to gastric emptying under the different dietary and Kwd+^®^ supplementation conditions. Casein bands: (1) α2-casein, (2) α1- and β-casein. Kwd+^®^ proteins: (1) actinidin, (2) thaumatin-like protein, (3) KiTH, (4) kirola (Act d11). Pea proteins: (1) lipoxygenase (LOX), (2) convicilin, (3) vicilin, (4) legumin α (11S), (5) vicilin (7S globulin), (6) legumin β (11S), (7) low-molecular-weight fragments (LMW, bands 1–4). NP, normoproteic diet; CHP, high-protein casein diet; PHP, high-protein pea diet. M, protein marker. Quantitative densitometric analysis of the principal protein fractions in casein-diets is provided in Supplementary Table S1.

To explore the mechanisms underlying the observed metabolic outcomes, gastric digestion patterns were analyzed by SDS-PAGE in chyme samples. In NP groups, Kwd+^®^ supplementation increased the degradation of casein subunits (Figure 1). Degradation of α2-casein reached 77 ± 15%, while degradation of the α1+β-casein fraction reached 90 ± 10% in the presence of Kwd+^®^. Under the CHP, the higher protein load produced a more intense banding pattern, but Kwd+^®^ supplementation further increased casein breakdown before stomach emptying. In this group, α2-casein degradation reached 86 ± 12%, while the α1+β-casein fraction reached 92 ± 6%, indicating enhanced early gastric proteolysis under high protein intake conditions (Table S1).

In PHP groups, high molecular weight pea protein storage proteins, including legumin α, vicilin, and convicilin, showed marked resistance to gastric digestion in unsupplemented animals, persisting as intact bands throughout the gastric incubation period. Kwd+^®^ supplementation in PHP animals resulted in the disappearance of these high molecular weight fractions, indicating actinidin-mediated degradation. However, lower molecular weight pea protein fractions persisted even with Kwd+^®^, confirming an enhanced but incomplete proteolysis (Figure 1, Table S2).

In contrast, analysis of intestinal content revealed uniform banding patterns across all groups regardless of protein type or Kwd+^®^ supplementation, with no residual intact dietary proteins even at high protein loading (Figure S3). This finding indicates that downstream pancreatic proteolysis efficiently completed the digestive process under all dietary conditions and confirms the stomach as the primary site of action of Kwd+^®^.

### 2.4. Plasma Amino Acid Profiles

Plasma concentrations of ten amino acids (alanine, glutamine, glutamate, threonine, glycine, histidine, tyrosine, valine, isoleucine, and leucine) and their sum were quantified at the end of the experimental period. After Bonferroni correction for multiple comparisons, no statistically significant effects of Kwd+^®^ were detected for any individual amino acid or for total amino acid levels.

Nevertheless, two amino acids displayed directional trends that may reflect biologically relevant shifts in digestive kinetics and nitrogen metabolism. Alanine showed opposing directional trends across dietary contexts: in the NP group, supplemented animals tended toward lower concentrations, whereas in CHP, supplemented animals tended toward higher concentrations. Although these contrasts did not remain significant after correction, both were associated with large standardized effect sizes (Table 3). For threonine, PHP animals supplemented with Kwd+^®^ tended toward higher circulating concentrations than unsupplemented PHP controls, suggesting a partial improvement in threonine bioavailability from pea protein facilitated by actinidin-enhanced proteolysis. This directional trend was likewise accompanied by a large effect size despite the lack of statistical significance after multiple-testing correction. No significant differences were detected for glutamate, glutamine, glycine, histidine, tyrosine, or BCAAs (i.e., valine, isoleucine, and leucine) after correction for multiple comparisons.

### 2.5. Plasma Protein Profile

**Figure 2:**
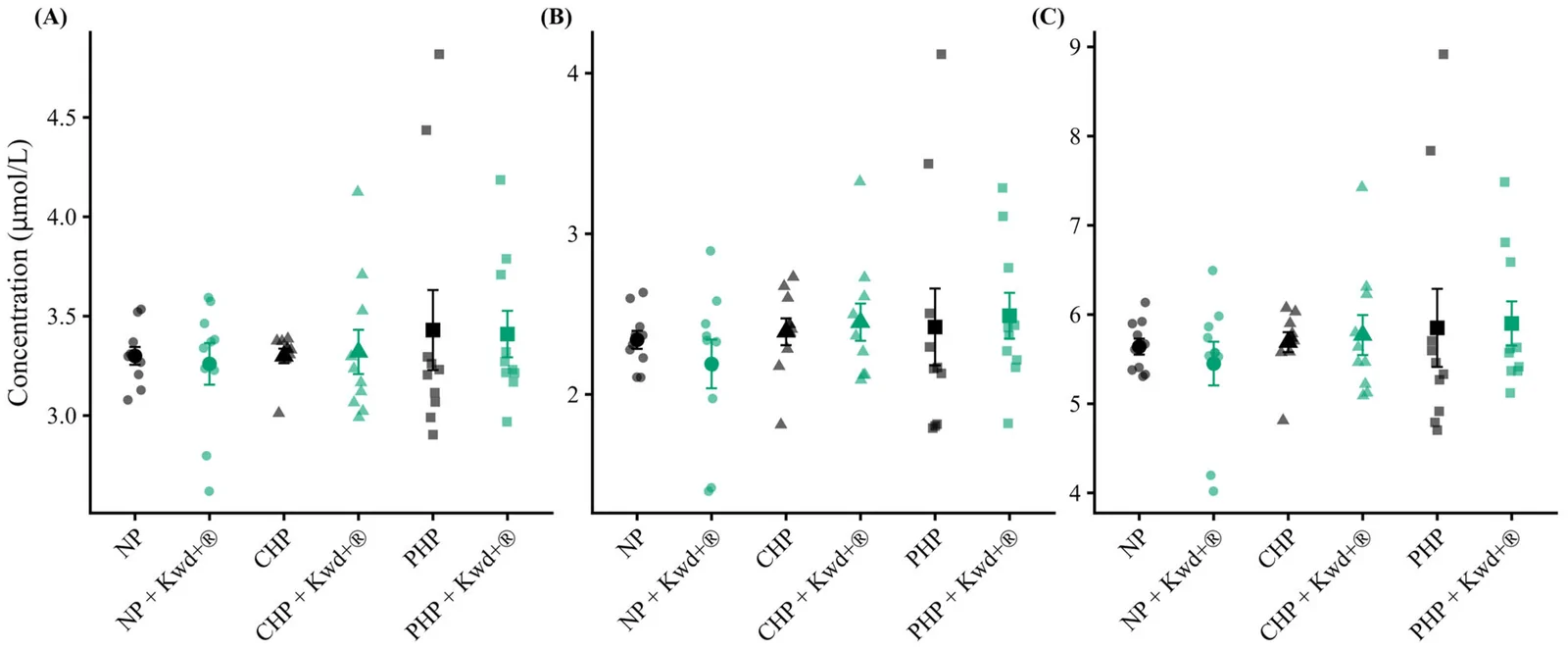
Plasma protein profiles across experimental dietary groups. (**A**) Albumin, (**B**) globulins, and (**C**) total plasma protein concentrations measured at the end of the intervention. Individual data points represent single animals, and larger symbols with error bars indicate group mean ± SEM. Black symbols represent control groups, whereas green symbols represent Kwd+^®^-supplemented groups. Circles correspond to NP diets, triangles to CHP diets, and squares to PHP diets. NP, normoproteic diet; CHP, high-protein casein diet; PHP, high-protein pea diet.

Beyond individual amino acid concentrations (Table S3) and consistent with the functional integration of amino acid availability at the systemic level, we examined circulating protein markers as a global readout of protein anabolic efficiency. Although total proteins, albumin, and globulins remained within physiological ranges across all groups (Figure 2A–C), PHP animals showed significantly reduced levels of all three markers compared with NP and CHP animals. Kwd+^®^ supplementation in PHP reversed this reduction, restoring plasmatic protein concentrations to levels comparable to those of CHP groups. No significant differences in plasma protein levels were detected between NP and NP-Kwd+^®^ or between CHP and CHP-Kwd+^®^.

### 2.6. Skeletal Muscle Fiber Morphology

**Figure 3:**
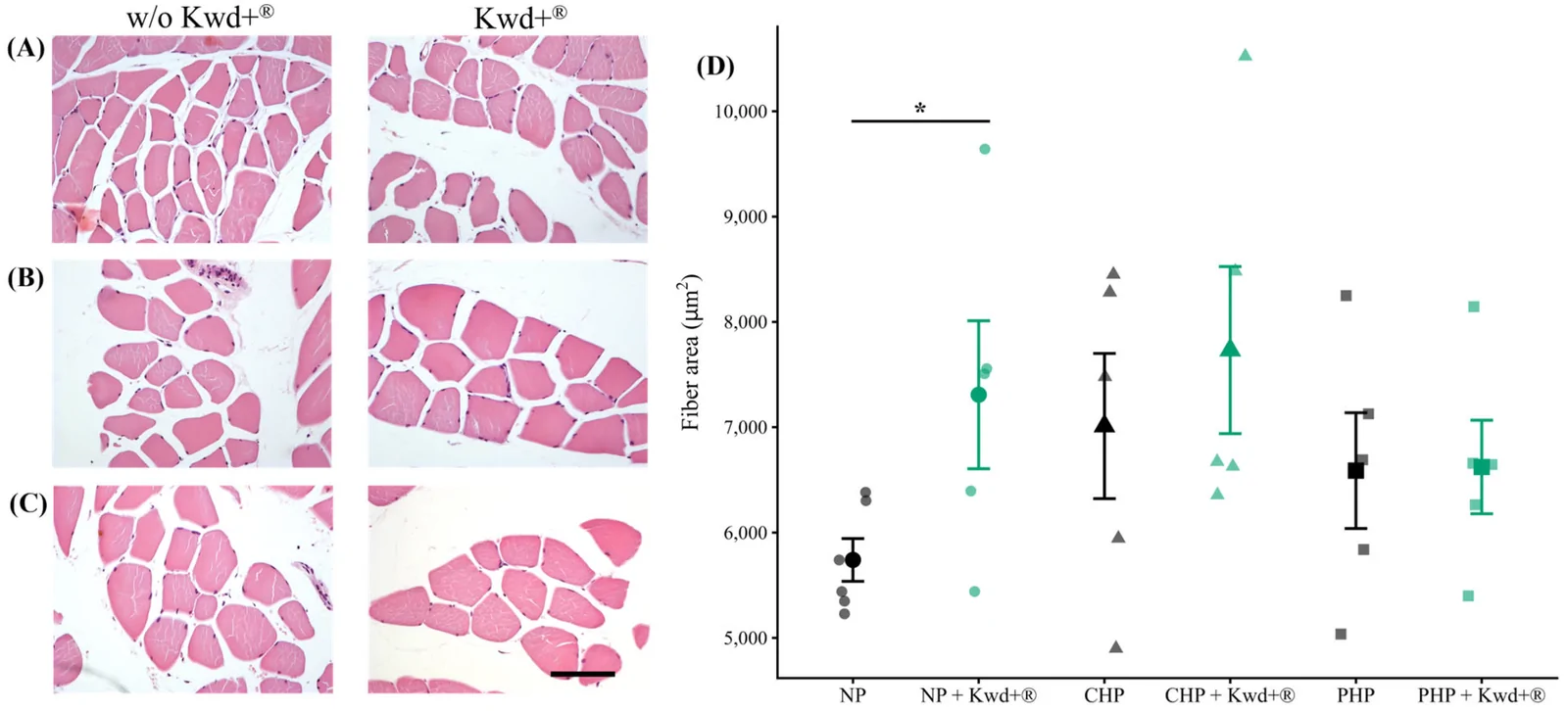
Representative hematoxylin–eosin–stained cross-sections of sural triceps muscle and quantification of muscle fiber cross-sectional area from animals fed the different experimental diets. (**A**) Normoproteic casein diet (NP), (**B**) ^®^high-protein casein diet (CHP), and (**C**) ^®^high-protein pea diet (PHP)^®^ (scale bar: 100 μm). (**D**) Individual data points represent single animals. Larger symbols with error bars indicate group mean ± SEM. Significant differences between groups are indicated by an asterisk (* *p* < 0.05). Black symbols represent control groups, whereas green symbols represent Kwd+^®^-supplemented groups. Circles correspond to NP diets, triangles to CHP diets, and squares to PHP diets.

In the NP diet, Kwd+^®^ supplemented animals exhibited significantly larger muscle fiber cross-sectional areas than unsupplemented controls (M = 7162.95, SD = 2949.15 µm^2^ vs. M = 4584.28, SD = 1674.26 µm^2^; mean difference = 2694.61, SE = 866.10, t(24) = 3.11, *p* = 0.005, d = 1.21, 95% CI [0.36, 2.03]) (Figure 3). This difference corresponded to a large effect size. No significant effects of Kwd+^®^ were detected in the CHP (*p* = 0.411) or PHP (*p* = 0.912) diets. The difference-of-differences contrast comparing the Kwd+^®^ effect in NP with that in PHP reached nominal significance (dd = 2598.90, SE = 1219.44, t(24) = 2.13, *p* = 0.044, d = 1.17, 95% CI [0.03, 2.28]), suggesting a larger Kwd+^®^ effect under the normoproteic diet; however, this result was no longer significant after Bonferroni correction (adjusted *p* = 0.088). The comparison between NP and CHP was not statistically significant.

### 2.7. Skeletal Muscle Contractile Properties and Fatigue Resistance

Given the pronounced hypertrophic effect of Kwd+^®^ under normoproteic conditions, contractile properties and fatigue resistance were assessed *in vivo* in NP and NP-Kwd+^®^ animals. Twitch contraction and relaxation kinetics, maximal twitch force, and the tetanic force–frequency relationship were fully preserved between groups, ruling out any pre-existing functional asymmetry and confirming an unchanged fiber type composition (Table S4; Figure S4).

**Figure 4:**
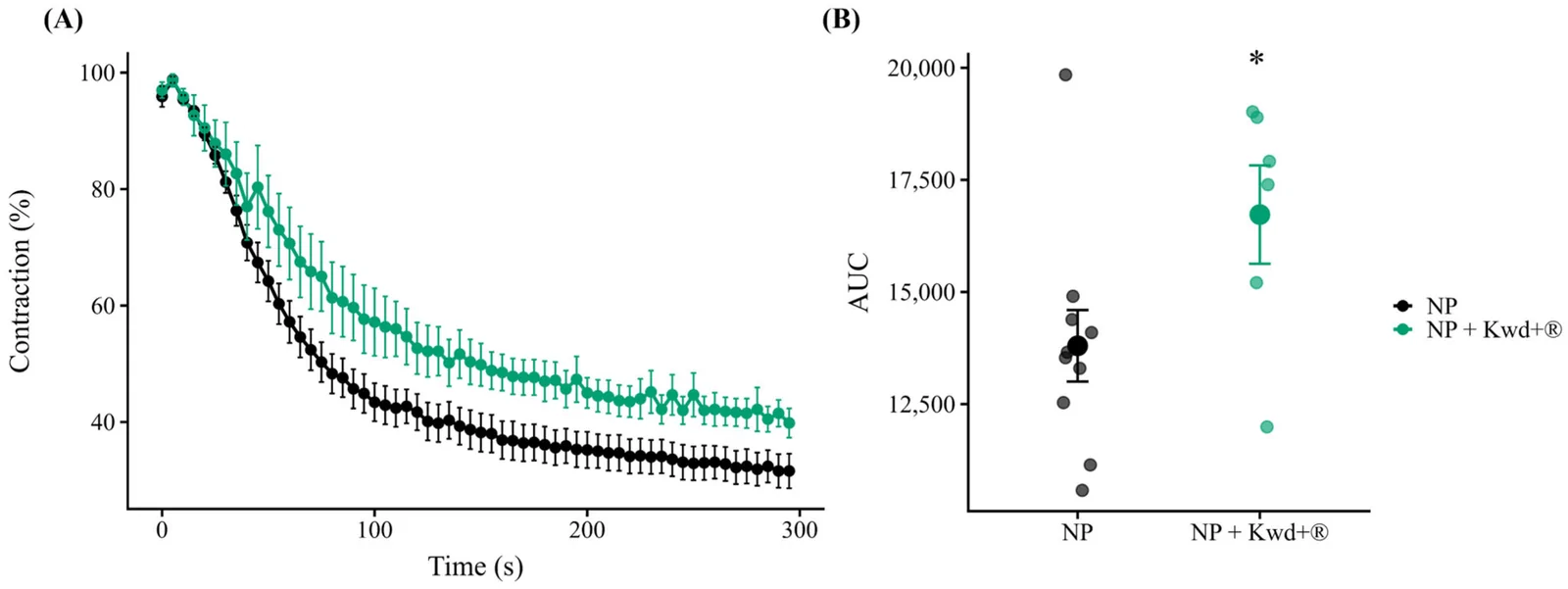
Effect of Kwd+^®^ supplementation on muscle fatigue resistance under normoproteic conditions. (**A**) Time course of relative muscle contraction during the fatigue protocol in isolated triceps surae muscles from NP and NP-Kwd+^®^ animals. Values represent mean ± SEM. (**B**) Global contractile performance during the fatigue protocol expressed as the area under the contraction–time curve (AUC). Each point represents an individual muscle, and black symbols indicate mean ± SEM. * *p* < 0.05 vs. NP.

The fatigue protocol revealed a clear functional divergence: NP-Kwd+^®^ muscles sustained markedly greater relative force throughout the stimulation period compared with unsupplemented NP (Figure 4A), resulting in a significantly greater global contractile performance quantified as the area under the contraction–time curve (Figure 4B), confirming superior fatigue resistance in the supplemented group. In addition, muscles from NP-Kwd+^®^ group exhibited lower post-fatigue maximal tetanic force loss (Figure S4), indicating a better post-fatigue recovery in animals supplemented with Kwd+^®^. The absence of differences in twitch kinetics indicates that this functional improvement was not driven by a shift in fiber type composition, but rather reflects metabolic and structural adaptations within the existing fiber type profile.

### 2.8. Plasmatic Biochemistry

Plasma biochemistry was assessed in NP and NP-Kwd+^®^ animals to characterise the metabolic context associated with the structural and functional muscle adaptations described above. GGT activity was significantly higher in NP-Kwd+^®^ compared with NP animals (1.34 ± 0.50 vs. 0.65 ± 0.42 U/L; t(18) = −3.33, *p* = 0.004), with all individual values remaining within the physiological reference range for the rat. No significant differences between groups were detected for ALT (55.8 ± 12.1 vs. 50.8 ± 6.5 U/L; *p* = 0.282), AST (185.7 ± 66.2 vs. 163.7 ± 56.2 U/L; *p* = 0.434), BUN (20.4 ± 4.4 vs. 21.0 ± 3.5 mg/dL; *p* = 0.714), or creatinine (0.98 ± 0.23 vs. 0.88 ± 0.16 mg/dL; *p* = 0.262). Plasma glucose was likewise comparable between groups (157.4 ± 32.8 vs. 157.3 +21.22 mg/dL; *p* = 0.99).

### 2.9. Myofibrillar Protein Content: Actin and Myosin Heavy Chain Expression

**Figure 5:**
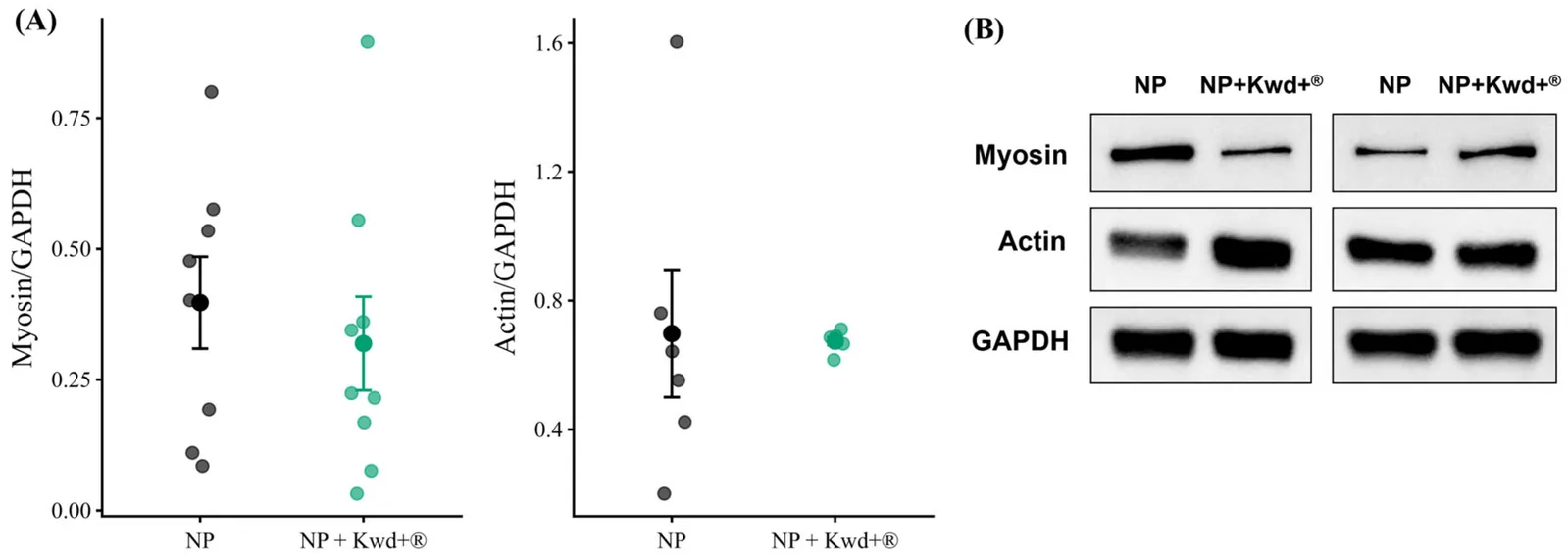
Myosin heavy chain and actin protein expression in triceps surae muscles from NP and NP-Kwd+^®^ animals. (**A**) Quantification of myosin heavy chain and actin protein levels in triceps surae homogenates from NP and NP-Kwd+^®^ groups determined by Western blot analysis. Protein abundance was normalized to GAPDH and expressed as relative protein levels. Individual points represent single animals and black symbols indicate mean ± SEM. (**B**) Representative Western blot images for myosin heavy chain, actin, and GAPDH from NP and NP + Kwd+^®^ muscles. Blots are representative of 6–9 independent muscle samples per group.

To further characterize the structural basis of the fiber-level adaptations observed in NP-Kwd+^®^ animals, total actin and myosin heavy chain content were quantified by Western blot in sural triceps homogenates from NP and NP-Kwd+^®^ groups (Figure 5). No significant differences were detected between groups for either protein. These results indicate that Kwd+^®^ supplementation did not modify the relative abundance of the major contractile proteins in sural triceps muscle.

## 3. Discussion

The present study shows that Kwd+^®^ modulates protein utilization in a diet-dependent manner, with distinct physiological responses depending on the protein source. CHP animals consumed considerably more total protein than NP rats, yet this did not translate into greater body mass accumulation. A similar pattern was observed in animals fed with PHP diet. This apparent paradox is well explained by the high thermic effect of protein, which dissipates a substantially larger fraction of ingested energy as heat compared to carbohydrates or fats and by the upregulation of amino acid oxidation and ureagenesis that occurs at supraphysiological protein intakes, effectively limiting net nitrogen retention [34,35]. Interestingly, within the PHP diet, Kwd+^®^-supplemented animals exhibited greater weight gain despite lower protein intake, suggesting that enhanced gastric protein digestion may improve the efficiency in which plant proteins are utilized despite being less digestible [31].

Regarding body composition, Kwd+^®^ supplementation reduced fat mass relative to protein intake in PHP animals, suggesting improved nutrient partitioning under plant-based hyperproteic conditions. In CHP animals, Kwd+^®^ increased the ratio of sural triceps mass to accumulated protein intake, indicating greater muscle accretion efficiency. Although whole-muscle mass did not differ between NP and NP-Kwd+^®^, this does not exclude biologically meaningful adaptations under normoproteic conditions, which may be better detected at the level of muscle fibre morphology [36,37,38]. Notably, both outcomes were associated with moderate-to-large effect sizes, supporting the biological relevance of these findings despite the absence of significant interaction effects.

Importantly, these effects cannot be attributed to differences in Kwd+^®^ exposure between diets, since supplementation was standardized relative to dietary protein content to maintain comparable enzymatic input across experimental conditions. Therefore, the observed physiological differences are more consistent with protein source-dependent responses to actinidin-mediated gastric proteolysis than with dose-related effects.

Fat deposition relative to accumulated protein intake and the ratio of sural triceps mass to protein intake were used as surrogate physiological indices of protein utilization efficiency. Accordingly, this concept should be interpreted within the context of these indirect measures rather than as a direct assessment of whole-body nitrogen retention. Classical measures of protein utilization, including nitrogen balance, urinary urea excretion and isotope-derived amino acid kinetics, were not evaluated in the present study.

To better understand the physiological responses observed, we examined gastric digestion of the different dietary proteins. Plant proteins generally exhibit lower gastric digestibility than animal proteins because of their compact structure and the presence of antinutritional factors [39]. Previous studies have shown that actinidin acts as a cysteine endopeptidase with a cleavage specificity largely complementary to pepsin, expanding the range of peptide bonds hydrolyzed during gastric digestion [22]. Recently, we demonstrated that Kwd+^®^ and pepsin share only 13–16% of predicted cleavage sites across different protein matrices, indicating that actinidin broadens rather than replaces endogenous gastric proteolysis [40]. Consistent with these findings, gastric chyme analysis showed greater degradation of casein proteins following Kwd+^®^ supplementation and enhanced fragmentation of high-molecular-weight pea proteins, although several lower-molecular-weight fractions remained detectable. These observations indicate that Kwd+^®^ enhances early gastric protein hydrolysis without completely overcoming the intrinsic resistance of pea storage proteins, a finding consistent with the persistence of low-molecular-weight peptide fractions observed after combined pepsin-Kwd+^®^ digestion *in vitro* [40].

These differences in gastric protein degradation may also contribute to the distinct feeding responses observed between diets: enhanced proteolysis in CHP with Kwd+^®^ could facilitate faster gastric processing and influence satiety signalling, whereas the persistence of partially intact proteins in PHP chyme may sustain gastric distension and prolong satiety, thereby limiting voluntary intake. However, because gastric emptying, satiety-related hormones and feeding regulation were not directly assessed, these physiological implications remain speculative. Overall, our findings support a partial improvement in the gastric digestibility of pea protein following Kwd+^®^ supplementation rather than a complete normalization to the digestion profile observed for casein [22,26,41].

These findings indicate that Kwd+^®^ acts primarily during the gastric phase of digestion by modifying the form and timing in which protein-derived nitrogen reaches the small intestine rather than by enhancing pancreatic digestion [42]. In this sense, analysis of intestinal content revealed uniform banding patterns across all groups regardless of protein type or Kwd+^®^ supplementation, with no residual intact dietary proteins even at high protein loading. In addition, this interpretation is further supported by *in vitro* evidence showing that Kwd+^®^ retains stable proteolytic activity across pH 3–5, a range over which pepsin activity declines markedly, thereby extending the functional hydrolytic window of gastric digestion, particularly during the early postprandial phase when luminal pH is transiently elevated [40].

Beyond the direct evidence of enhanced gastric proteolysis, our findings suggest that the physiological relevance of Kwd+^®^ may lie in modifying the kinetics of protein digestion rather than the final extent of protein degradation. By accelerating the breakdown of intact dietary proteins, actinidin may alter the timing and molecular form in which protein-derived nitrogen reaches the small intestine. Although nutrient sensing and luminal peptide profiles were not evaluated in the present study, such kinetic effects could contribute to differences in protein utilization even when intestinal digestion is complete and terminal plasma amino acid concentrations remain unchanged [26,27].

Plasma amino acid profiling revealed only limited differences between dietary groups after correction for multiple comparisons, indicating that Kwd+^®^ supplementation did not substantially alter systemic amino acid availability under the experimental conditions. Although alanine and threonine showed directional trends with moderate-to-large effect sizes, these differences were not statistically significant after Bonferroni correction and should therefore be interpreted cautiously. Alanine is the primary vehicle for nitrogen shuttling between skeletal muscle and liver, and the directional shifts observed in the NP and CHP groups may reflect subtle changes in interorgan nitrogen trafficking associated with differences in protein digestion kinetics. Threonine, required for protein synthesis and additionally supports gut integrity, immune function, and hepatic lipid homeostasis, showed a tendency toward higher concentrations in PHP-Kwd+^®^ animals, a pattern consistent with a partial improvement in plant protein bioavailability facilitated by actinidin-mediated gastric proteolysis. In addition, the absence of significant differences in histidine, tyrosine and branched-chain amino acids, key substrates for neurotransmitter production and muscle protein anabolism, further supports the interpretation that enhanced gastric proteolysis does not necessarily translate into sustained increases in circulating amino acid concentrations. Rather, changes in digestive kinetics may modify the temporal profile of amino acid appearance without substantially altering terminal plasma concentrations, which represent only a single time point of a dynamic postprandial process. If amino acids released more rapidly from the gastric compartment were efficiently directed toward tissue utilization, plasma concentrations could remain unchanged even under conditions of improved digestive efficiency, as uptake would pace the rate of appearance. Overall, the amino acid profile is more consistent with modest, kinetics-driven changes in amino acid release than with major alterations in systemic amino acid availability, a conclusion that also agrees with the complete intestinal digestion observed across all experimental groups [43,44,45].

Consistent with this interpretation, circulating protein markers may better reflect the cumulative physiological impact of improved digestion. PHP animals showed lower concentrations of total proteins, albumin and globulins than NP and CHP animals, whereas these markers were restored by Kwd+^®^ supplementation. This finding is consistent with the lower digestibility typically associated with plant proteins and with the enhanced gastric proteolysis observed following Kwd+^®^ supplementation [20,45]. Because these proteins integrate systemic protein status over longer time periods than individual amino acids, their normalization in PHP-Kwd+^®^ may provide stronger evidence of improved protein utilization than terminal plasma amino acid concentrations alone.

Taken together, the differences in gastric proteolysis, circulating amino acid patterns, and plasma protein markers described above raise an important functional question: whether improvements in protein digestibility translate into measurable skeletal muscle adaptations. Cross-sectional area analysis of sural triceps fibers revealed the largest values in Kwd+^®^ supplemented NP animals, suggesting that improved gastric protein digestion may contribute to enhanced muscle remodeling under normoproteic conditions. Importantly, the increase in fiber cross-sectional area in NP-Kwd+^®^ was associated with a large effect size.

Regarding hyperproteic diets, despite substantially higher protein intake, CHP animals did not achieve greater muscle fiber cross-sectional areas than NP-Kwd+^®^. This observation is consistent with the concept that muscle protein synthesis reaches a saturation threshold beyond which additional amino acid supply provides little further anabolic benefit. PHP diets similarly failed to increase muscle fiber area despite high protein loads, which aligns with previous reports indicating that plant proteins often stimulate muscle protein synthesis less efficiently than animal proteins due to their lower digestibility [45,46,47]. Although Kwd+^®^ partially improved gastric degradation of pea proteins, this effect was insufficient to fully overcome the intrinsic structural constraints associated with plant protein matrices. Therefore, the clearest morphometric effect of Kwd+^®^ was observed under normoproteic conditions, while the evidence for a statistically stronger response in NP than in other diets remains suggestive rather than definitive.

Collectively, these findings suggest that the anabolic potential of Kwd+^®^ is most clearly expressed when dietary protein intake operates within the physiological range that maximally stimulates muscle protein synthesis rather than under supraphysiological protein loads. This context-dependent response represents one of the principal findings of the present study.

The mechanisms underlying the hypertrophic response in NP-Kwd+^®^ animals remain to be established. Although threonine showed a directional increase in this group, this difference was not statistically significant after correction for multiple testing. Therefore, any involvement of amino acid-dependent anabolic signalling pathways, including mTORC1, remains speculative and should be addressed in future studies through direct molecular analyses. In addition, the pronounced hypertrophic effect of Kwd+^®^ supplementation under normoproteic conditions prompted us to examine whether these structural adaptations were accompanied by functional changes in muscle performance. Therefore, contractile properties and fatigue resistance were assessed in the normoproteic groups (NP and NP-Kwd+^®^). *In vivo* assessment of the sural triceps contraction revealed fully preserved twitch kinetics, maximal twitch force, and an identical tetanic force-frequency relationship between NP control and NP-Kwd+^®^, ruling out any pre-existing functional asymmetry and suggesting an unchanged fiber type composition, as contractile speed and calcium handling are predominantly determined by the myosin heavy chain isoform profile [48]. The similar expression of total actin and myosin heavy chain content in sural triceps homogenates from NP and NP-Kwd+^®^ animals further supported this interpretation. The stable contractile protein ratios, in the context of the larger fiber cross-sectional area observed in NP-Kwd+^®^, suggest a proportional expansion of the myofibrillar apparatus consistent with genuine myofibrillar hypertrophy rather than sarcoplasmic expansion [49]. The fatigue protocol, however, revealed that NP-Kwd+^®^ muscles sustained markedly greater relative force throughout the stimulation period, with significantly lower post-fatigue tetanic force loss, confirming superior fatigue resistance [50]. The absence of differences in twitch kinetics further supports the interpretation that these functional improvements were not driven by shifts in muscle fiber type composition. Collectively, these results indicate that Kwd+^®^ supplementation under NP conditions promotes coordinated structural and functional adaptations in skeletal muscle, resulting in a phenotype characterized by increased fiber size and improved resistance to fatigue [50,51]. These adaptations are consistent with improved protein utilization without evidence of changes in intrinsic contractile phenotype.

It is remarkable that the improved fatigue resistance observed in NP-Kwd+^®^ animals was accompanied by higher serum GGT levels, suggesting an elevated glutathione turnover, although all values remained within the physiological range. The absence of significant differences in ALT, AST, BUN, or creatinine between groups argues against a systemic catabolic or inflammatory explanation for the elevated GGT and supports the interpretation that this difference reflects upregulated glutathione recycling capacity rather than tissue damage. Plasma glucose was likewise comparable between groups, providing no evidence of altered glycaemic status. Nonetheless, it is mechanistically plausible that a more efficient redox environment, combined with larger fibre cross-sectional area could facilitate glycogen sparing and reduce fatigue during sustained contractile activity in NP-Kwd+^®^ animals [52,53,54]. Because no direct measurements of oxidative status, glycogen content or antioxidant capacity in skeletal muscle were performed, the physiological significance of this finding cannot be established from the present data. Future studies incorporating markers of oxidative metabolism and muscle energy stores will be required to clarify the mechanisms underlying the improved fatigue resistance [50,55,56].

Several mechanistic questions raised by the present findings warrant further research. Future studies should determine whether the enhanced gastric proteolysis induced by Kwd+^®^ modifies luminal peptide profiles and downstream nutrient sensing. The molecular mechanisms underlying the hypertrophic response, including the possible involvement of mTORC1 signalling, should also be evaluated by direct analysis of anabolic signalling pathways Finally, the mechanisms responsible for the improved fatigue resistance observed in NP-Kwd+^®^ animals remain to be established through direct assessment of muscle energy metabolism and oxidative status. Addressing these questions would help clarify how enhanced gastric protein digestion translates into improved protein utilization and skeletal muscle adaptation.

## 4. Material and Methods

### 4.1. Experimental Design

Sixty male Wistar rats (mean body weight: 333.5 ± 7.6 g) were obtained from a certified supplier and housed at the Animal Facility of the Universidad Autónoma de Madrid (Registration number ES-28079-0000097) in compliance with the European Commission Directive 86/609/CEE and the Guide for the Care and Use of Laboratory Animals (NIH Publication No. 85-23, revised 1996). All procedures were approved by the Ethics Committee of the Universidad Autónoma de Madrid and the Comunidad Autónoma de Madrid (PROEX 156.5/24).

Animals were maintained under controlled environmental conditions: temperature 22 ± 1 °C, relative humidity 50–60%, ventilation rate of 15–20 air changes per hour, and a 12 h light/dark cycle. Animals were housed in pairs in polysulfone cages (type III, SODISPAN; floor area 840 cm^2^, height 15 cm) containing standard wood-shaving bedding (SAFE^®^ 3–4S, Lignocel, J. Rettenmaier & Söhne GmbH + Co. KG, Rosenberg, Germany), with *ad libitum* access to food and water. Environmental enrichment was provided in the form of cardboard tubes, sizzle pads, and wood blocks. An acclimation period of 7 days was allowed prior to the start of the experimental interventions.

Rats were randomly assigned to one of six experimental groups (n = 10 per group) and subjected to their respective dietary interventions for 8 weeks: (1) normoproteic casein-based diet (18% protein; NP); (2) normoproteic casein diet supplemented with Kwd+^®^ (6.3% *w*/*w* of the diet; NP-Kwd+^®^); (3) high-protein casein diet (30% protein; CHP); (4) high-protein casein diet supplemented with Kwd+^®^ (10.5% *w*/*w* of the diet; CHP-Kwd+^®^); (5) high-protein pea protein diet (30% protein; PHP); and (6) high-protein pea protein diet supplemented with Kwd+^®^ (10.5% *w*/*w* of the diet; PHP-Kwd+^®^). The inclusion level of Kwd+^®^ was adjusted according to the protein content of each diet in order to maintain a consistent enzyme-to-protein ratio across experimental conditions, thereby ensuring comparable proteolytic exposure despite differences in total protein intake. A one-way ANOVA and an independent-samples *t* test were conducted to assess baseline weight differences among diets and between Kwd+^®^ groups, respectively. No statistically significant differences were found either among diets (F(2, 57) = 1.21, *p* = 0.306) or between Kwd+^®^ groups (t(58) = 0.29, *p* = 0.776). Diets were formulated and supplied by Inotiv (normoproteic reference: TD.96180; high-protein reference: TD.91361). Kwd+^®^, an actinidin-enriched kiwifruit concentrate, was provided by Pharmactive Biotech Products, S.L.U. (Madrid, Spain).

The Kwd+^®^ batch used in the present study exhibited a proteolytic activity of 21,000 PA/g product, determined using a casein-based assay (2% casein, 40 °C, 10 min) according to methodologies commonly used in the literature [57,58]. Kwd+^®^ contained 6% protein, of which 50–60% corresponded to actinidin as estimated by SDS–PAGE densitometric analysis. Previous studies have reported that actinidin remains active across a broad pH range compatible with gastric digestion, with optimal activity between 35 and 40 °C [18]. During feed preparation, processing conditions were controlled to minimize enzymatic inactivation, and the material was not exposed to temperatures above 30 °C for more than 10 min.

### 4.2. Animal Evolution

Body weight and food intake were recorded weekly throughout the 8-week intervention period. Food consumption was measured at the cage level; individual intake was estimated by dividing cage consumption by two. The day before euthanasia, animals were fasted overnight. On the following morning, animals were given access to their respective diet for 30 min, after which the remaining chow was removed and weighed to calculate acute food intake. Glucose was also measured using a Contour^®^ Next Ascentia Diabetes Care glucometer (Basel, Switzerland).

### 4.3. In Vivo Skeletal Muscle Contractile Function

Motor unit contractile properties and rate of force development were assessed *in vivo* in the medial sural triceps, adapting the protocol described by Wasicki et al. [59]. Briefly, animals were deeply anesthetized with isofluorane (induction: 5%; maintenance: 2.5%) and kept in prone position. The sural triceps was surgically isolated and connected via the Achilles tendon to a force transducer, in turn connected to a mp30 BIOPAC System (BIOPAC Systems, Inc., Goleta, CA, USA). Isometric force was recorded with the muscle at an optimal passive tension of 20 mN. Stimulation electrodes were attached to the muscle, and rectangular pulses were applied (0.1 ms, ≤0.5 V) to stabilize muscle function. Then, the supramaximal voltage was first determined by progressively increasing stimulus intensity (0.5 ms square pulses, 1 Hz) until no further increase in contractile response was observed, and this voltage was held constant throughout the experiment. From the twitch recording, maximal force, contraction and half contraction times, maximal and medium (25–75%) contraction slopes, relaxation and half relaxation times, and maximal and medium (25–75%) relaxation slopes were determined. Afterwards, tetanic force was assessed by delivering 200 ms stimulus trains at frequencies increasing from 20 to 100 Hz, in 10 Hz increments, with a 30 s inter-train interval; the maximum force recorded was taken as the optimal tetanic tension. The fatigue protocol consisted of 60 tetanic stimulus trains at 60 Hz, each train lasting 2 s followed by a 3 s rest period. Afterwards, a post-fatigue tetanic stimulus was applied.

### 4.4. Animal Euthanasia and Sample Collection

After skeletal muscle assays, rats were euthanized by inhalatory anesthesia overdose (isoflurane, 5%) followed by exsanguination. Blood samples were collected in EDTA-covered tubes and centrifuged (2000× *g*, 10 min, 4 °C) to obtain plasma samples, which were stored at −80 °C until use. The body cavity was then opened and the stomach and small intestine were removed. Stomach chyme and small intestine contents were extracted and stored separately at −80 °C until analysis. In addition, visceral and epidydimal fat pads were dissected and weighed together. Unstimulated sural triceps was also dissected, weighed, and divided into three sections, two of them were frozen at −80 °C, and one was fixed in paraformaldehyde (10%).

### 4.5. SDS-PAGE Electrophoresis

Gastric and intestinal protein degradation patterns were assessed by SDS–PAGE electrophoresis. Proteins were extracted from thawed gastrointestinal contents by homogenizing approximately 800 mg of sample with 10 mL of extraction buffer (12.5 mM sodium tetraborate, 2% SDS) using an Ultra-Turrax homogenizer (IKA-Werke GmbH & Co. KG, Staufen im Breisgau, Germany). Homogenates were incubated for 1 h at room temperature with gentle agitation and centrifuged at 4700 rpm for 10 min to recover the soluble protein fraction [29].

For electrophoretic analysis, protein extracts were mixed with 5× SDS–PAGE sample loading buffer (NZYtech, Lisbon, Portugal) and adjusted to 1× final concentration, heated at 100 °C for 10 min, and centrifuged at 3500× *g* for 3 min. Aliquots of 15–30 μL were loaded onto precast gradient SDS–PAGE gels (4–12%, NZYtech) and electrophoresed according to the manufacturer’s instructions. Gels were stained with Coomassie Brilliant Blue R-250 (Sigma, St. Louis, MO, USA) to visualize protein bands. A prestained molecular weight marker (NZYColour Protein Marker II, NZYtech) was loaded in parallel lanes to estimate protein molecular masses.

Gel images were acquired after staining and band intensities were quantified by densitometry using ImageJ software (version 1.53, National Institutes of Health, Bethesda, MD, USA). Band density was expressed in arbitrary density units (ADU) to compare protein degradation patterns among dietary treatments. For the principal casein fractions, degradation was additionally expressed as percentage reduction in band intensity relative to the corresponding untreated control bands. Control conditions were defined as 100% band intensity. Due to the digestion patterns observed in pea protein diets, degradation was evaluated qualitatively based on band appearance, disappearance, and/or persistence.

### 4.6. Nuclear Magnetic Resonance (NMR) Spectroscopy

Plasma concentrations of amino acids and proteins were quantified by proton nuclear magnetic resonance (^1^H-NMR) spectroscopy using the validated metabolomics platform of Biosfer Teslab Advancing Testing (Barcelona, Spain), following their standard operating procedures. Plasma samples were analyzed using an automated NMR-based metabolomics workflow designed for the quantitative profiling of low molecular weight metabolites in biological fluids. The following amino acids were included in the quantification panel: alanine, glutamine, glutamate, threonine, glycine, histidine, tyrosine, valine, isoleucine, and leucine. A composite variable representing total amino acid concentration was calculated as the arithmetic sum of the ten individual amino acid concentrations.

### 4.7. Plasmatic Biochemistry Measurements

Circulating protein status (total proteins, albumin and globulins), blood ureic nitrogen (BUN), creatinine, GGT, ALT and AST were measured using a PointCare V3 Automatic Chemistry Analyzer (Tianjin MNCHIP Technologies Co., Ltd., Tianjin, China). Samples were processed following the manufacturer’s instructions using a General Panel+ kit (ref. MN00014, RAL Laboratories, Sant Quirze del Vallès, Barcelona, Spain).

### 4.8. Histology

Sural triceps samples were fixed in 10% formaldehyde and embedded in paraffin. Sections of 5 μm were made using a microtome and then stained with haematoxylin and eosin. A minimum of 15 random fields were photographed for each sample under a 20× objective with a Zeiss Axioskop 2 microscope equipped with the image analysis software package LAS X (Leica, Munich, Germany). The area of each muscle fiber was calculated using the Image J-Fiji program 2.16.

### 4.9. Western Blot

Western blot analysis was performed as previously described [60]. Briefly, frozen fragments of sural triceps were homogenized in RIPA buffer (Tris-HCl 50 mM (pH 7.4); NaCl 150 mM; NP-40 1%; Sodium deoxicolate 0.5%; SDS 0.1% and EDTA 1 mM) using a TissueLyser II Beadmill homogenizer (QIAGEN GmbH, Hilden, Germany). 40 µg protein were loaded in each well, and were separated by electrophoresis for their molecular weight in gradient SDS-polyacrylamide gel (Bio-Rad Laboratories, Hercules, CA, USA). Membranes were incubated with either Anti-Alpha Skeletal Muscle Actin antibody [EPR18430] (rabbit polyclonal, 1:2000; Abcam, Cambridge, UK) or Myosin Heavy Chain Antibody (mouse monoclonal; 1:2000; Biotechne-R&D Systems, Minneapolis, MN, USA). Appropriate secondary antibodies (1:1000, GE Healthcare Systems, Chicago, IL, USA) were used. The membranes were developed using a Chemidoc Imaging System, and images were quantified using Image Lab Software (version 6.1, Bio-Rad Laboratories, Hercules, CA, USA). The same membrane was used to correct protein expressions in each sample, by means of a monoclonal antibody mouse anti GAPDH (1:10,000, Applied Biosystems/Ambion, Austin, TX, USA).

### 4.10. Statistical Analysis

All statistical analyses were performed using SPSS v.25 (IBM Corp., Armonk, NY, USA) and R (v4.5.2).

An initial exploratory analysis was conducted for selected outcomes using classical ANOVA and Student’s *t*-test approaches. Physiological and biochemical variables are expressed as mean ± SEM throughout. Body weight gain and weight gain per gram of protein intake were compared between experimental groups across time intervals using unpaired two-way ANOVA, with diet and Kwd+^®^ supplementation as between-subject factors, followed by Bonferroni post hoc tests. Plasma biochemical variables were compared between experimental groups using one-way ANOVA followed by Bonferroni post hoc tests.

Analyses of muscle contractile properties were restricted to comparisons between the NP and NP-Kwd+^®^ groups. Twitch contractile parameters were compared between groups using an unpaired Student’s *t*-test. Tetanic force and muscle fatigue responses were analyzed using unpaired two-way ANOVA, with supplementation and fatigue state as factors. The effect of Kwd+^®^ supplementation on the transition between pre- and post-fatigue tetanic force was further examined using a paired two-way ANOVA. The overall contractile response across the force–frequency relationship in tetanic contraction was summarized by calculating the differences in area under the force–frequency curve (dAUC), and differences between NP and NP-Kwd+^®^ groups were assessed using an unpaired Student’s *t*-test. Western blot relative expression values were first analysed using a Saphiro–Wilks test to identify outliers, and afterwards groups were compared using an unpaired Student’s *t*-test.

For the remaining outcomes, a more comprehensive modelling strategy was applied. For each outcome, the effects of diet (NP, CHP, PHP), Kwd+^®^ supplementation (control vs. Kwd+^®^), and their interaction were initially examined within a mixed-model framework with cage as a random intercept, given that the intervention was assigned at cage level (two animals per cage). When between-cage variance was negligible, the random effect was removed [61]. Model assumptions were evaluated at both the individual-residual and, where applicable, the random-effect level. When residuals deviated from normality, aligned rank transform (ART) ANOVA was used instead [62]. When heteroscedasticity was detected, candidate residual variance structures (by diet, by Kwd+^®^, by their additive combination, and by design cell) were fitted and formally compared via likelihood ratio tests under maximum likelihood (ML), with model selection guided by AIC and BIC; the retained structure was re-estimated under restricted maximum likelihood (REML) for final inference.

Planned contrasts, derived from estimated marginal means (EMM), addressed two a priori questions [63,64,65,66]: (a) the simple effect of Kwd+^®^ within each diet and (b) difference-of-differences contrasts comparing the Kwd+^®^ effect in NP with that in CHP and PHP. Omnibus effect sizes were reported as partial eta-squared (η^2^p) with 95% confidence intervals (CI). Pairwise contrasts were quantified as standardized mean differences (*d*) with 95% confidence intervals; under heterogeneous variances, the denominator was the group-specific residual SD or the root-mean-square of the SDs of the compared groups. Bonferroni correction was applied per family of contrasts within each outcome: simple effects of Kwd+^®^ within each diet involved a single comparison per family (m = 1) and required no adjustment; difference-of-differences contrasts were adjusted for two comparisons (adjusted α = 0.025). Statistical significance was set at α = 0.05 unless otherwise stated.

The outcome-specific details below describe only what departs from the general strategy outlined above (Figure S1).

*Body weight gain*. Weight gain per cage was recorded across four consecutive intervals (weeks 0–2, 2–4, 4–6, 6–8; 30 cages × 4 intervals). Temporal dependence was modeled with an unstructured (UN) covariance matrix for intervals nested within cages. Fixed effects included diet, Kwd+^®^, interval, cumulative protein intake standardized within each diet as a covariate, and all two- and three-way interactions. Marginal means were evaluated at three levels of protein intake within each diet (−1 SD, mean, +1 SD); simple effects of Kwd+^®^ were therefore adjusted for three comparisons (adjusted α = 0.017) and difference-of-differences contrasts for two comparisons within each intake level (adjusted α = 0.025).

*Plasma amino acids.* Ten amino acids and a total indicator (sum of the ten) were quantified at the end of the experiment. To control the family-wise error rate across the 11 simultaneous variables, significance was set at α = 0.0045 (Bonferroni, 0.05/11).

*Fat deposition*. The outcome was the total fat/accumulated protein ratio.

*Unstimulated muscle mass.* The outcome was the unstimulated triceps mass/accumulated protein ratio.

*Muscle fiber area.* The outcome was the cross-sectional fiber area (µm^2^), with multiple fibers measured per rat (23–66 per animal; 1102 observations). The initial model included random intercepts for both cage and rat; cage-level variance was negligible, so only the rat-level intercept was retained.

## 5. Conclusions

In summary, supplementation with the actinidin-enriched kiwifruit concentrate Kwd+^®^ enhanced gastric protein hydrolysis and modulated protein utilization in a diet-dependent manner. While circulating amino acid concentrations remained largely unchanged, Kwd+^®^ supplementation improved markers of protein utilization efficiency, including reduced fat deposition in animals fed a high-protein pea diet and increased muscle protein efficiency in animals receiving a high-protein casein diet. Under normoproteic conditions, Kwd+^®^ supplementation was associated with a larger skeletal muscle fiber cross-sectional area and improved fatigue resistance without alterations in fiber type composition or contractile protein content. These findings suggest that enhancing early gastric proteolysis through actinidin may improve the efficiency of dietary protein utilization at the tissue level.

The present study demonstrates that Kwd+^®^ supplementation produces protein source- and intake-dependent effects on protein digestibility, metabolic handling, and skeletal muscle physiology, highlighting the importance of digestive kinetics as a modulator of protein utilization efficiency beyond total protein intake. Importantly, these findings indicate that improving early-phase protein digestion does not necessarily translate into increased circulating amino acid concentrations but rather into a more efficient partitioning of dietary nitrogen toward functional tissue outcomes such as muscle accretion and reduced adiposity. This supports the concept that the temporal dynamics of amino acid delivery, rather than absolute amino acid availability, may represent a key determinant of anabolic efficiency under physiological conditions.

From a nutritional perspective, these results suggest that digestive enhancement strategies may be particularly relevant under conditions in which protein utilization is suboptimal, such as plant-based diets or situations of limited effective protein intake.

Although the experiment included 60 rats distributed across six experimental groups, the resulting sample size of n = 10 animals per group may have limited statistical sensitivity, particularly for detecting small-to-moderate effects within a 3 × 2 factorial design. In addition, because diet and Kwd+^®^ supplementation were assigned at cage level, the effective number of independent experimental units was smaller for outcomes measured or aggregated at the cage level. Accordingly, non-significant findings should be interpreted cautiously and should not be considered definitive evidence of absence of biological effect. Rather, the present results should be viewed as preclinical evidence providing effect-size estimates and mechanistic indications to guide future confirmatory studies with larger sample sizes and direct assessments of protein utilization kinetics.

## Figures and Tables

**Table 1:** Effects of Kwd+^®^ Supplementation on the Fat Mass-to-Accumulated Protein Ratio.

**Panel A. Simple effects of Kwd+^®^ within each diet**							
**Diet**	**Control Mean (SD)**	**Kwd+^®^ Mean (SD)**	**Mean Difference**	**SE**	**t(df)**	***p***	**d [95% CI]**
NP	0.10 (0.03)	0.09 (0.03)	−0.0144	0.0129	−1.12 (18)	0.280	−0.50 [−1.38, 0.40]
CHP	0.05 (0.01)	0.06 (0.01)	0.0019	0.0059	0.32 (18)	0.757	0.14 [−0.74, 1.02]
PHP	0.06 (0.01)	0.05 (0.01)	−0.0108	0.0049	−2.19 (18)	0.042	−0.98 [−1.90, −0.04]
**Panel B. Difference-of-differences contrasts**							
**Contrast**		**dd**		**SE**	**t(df)**	***p***	**d [95% CI]**
NP vs. CHP		−0.0163		0.0142	−1.15 (25.1)	0.263	−0.72 [−1.97, 0.54]
NP vs. PHP		−0.0036		0.0138	−0.26 (23.2)	0.797	−0.16 [−1.40, 1.08]

Note. M (Mean) and SD (Standard Deviation) are sample descriptive statistics. dd = difference in simple Kwd+^®^ effects (NP minus comparison diet); positive values indicate a larger Kwd+^®^ effect in NP. NP, normoproteic diet; CHP, high-protein casein diet; PHP, high-protein pea diet. SE, standard error. CI, confidence interval.

**Table 2:** Effects of Kwd+^®^ Supplementation on the Unstimulated Triceps Mass-to-Accumulated Protein Ratio.

**Panel A. Simple effects of Kwd+^®^ within each diet**							
**Diet**	**Control M (SD)**	**Kwd+^®^ M (SD)**	**Diff**	**SE**	**t(df)**	***p***	**d [95% CI]**
NP	0.0121 (0.0018)	0.0131 (0.0017)	0.0010	0.0008	1.20 (24)	0.241	0.57 [−0.38, 1.51]
CHP	0.0070 (0.0006)	0.0084 (0.0008)	0.0014	0.0004	3.50 (24)	0.002	2.16 [0.79, 3.49]
PHP	0.0098 (0.0008)	0.0101 (0.0010)	0.0003	0.0005	0.66 (24)	0.516	0.37 [−0.74, 1.48]
**Panel B. Difference-of-differences contrasts**							
**Contrast**		**dd**		**SE**	**t(df)**	***p***	**d [95% CI]**
NP vs. CHP		−0.0004		0.0009	−0.49 (24)	0.627	−0.35 [−1.72, 1.04]
NP vs. PHP		0.0006		0.0009	0.69 (24)	0.494	0.49 [−0.90, 1.86]

Note. M (Mean) and SD (Standard Deviation) are sample descriptive statistics. dd = difference in simple Kwd+^®^ effects (NP minus comparison diet); positive values indicate a larger Kwd+^®^ effect in NP. NP, normoproteic diet; CHP, high-protein casein diet; PHP, high-protein pea diet. SE, standard error. CI, confidence interval.

**Table 3:** Simple Effects of Kwd+^®^ Supplementation on Plasma Amino Acid Concentrations (μmol/L).

Amino Acid	Diet	Control M (SD)	Kwd+^®^ M (SD)	t(df)	*p*	*p* ^a^	d [95% CI]
Alanine	NP	672.06 (63.65)	626.39 (180.50)	2.03 (51)	0.048	0.528	0.96 [−0.01, 1.93]
	CHP	576.70 (73.38)	688.99 (123.63)	−2.42 (51)	0.019	0.209	−1.08 [−2.01, −0.16]
Threonine	PHP	234.15 (56.96)	306.50 (69.54)	−2.53 (50)	0.014	0.154	−1.16 [−2.12, −0.21]

Note. Contrasts reflect the simple effect of Kwd+^®^ within each diet (control—Kwd+^®^). M (Mean) and SD (Standard deviation) are sample descriptive statistics in the original metric; inferential statistics (t, *p*, d) derive from aligned rank transform (ART) ANOVA on ranked data. NP, normoproteic diet; CHP, high-protein casein diet; PHP, high-protein pea diet. ^a^ Bonferroni-adjusted *p* value (adjusted α = 0.0045; 0.05/11 amino acid outcomes).

## Data Availability

All data generated or analysed during this study are included in this published article and its Supplementary Information files. Raw data are available on demand.

## Supplementary Materials

The following supporting information can be downloaded at https://www.mdpi.com/article/10.3390/ijms27167086/s1.
