# Supplementary material for: Actinidin-Enriched Kiwifruit Concentrate (Kwd+®) Improves Protein Utilization Efficiency and Modulates Body Composition and Muscle Function in a Protein Source-Dependent Manner
Source: Int J Mol Sci. 2026 Aug 7;27(16):7086. doi: 10.3390/ijms27167086 (PMC13513624; doi:10.3390/ijms27167086)
Supplement: Supplementary file 1 [file ijms-27-07086-s001.zip › ijms-4274565-supplementary.pdf]

Actinidin-Enriched Kiwifruit Concentrate (Kwd+®) Improves Protein Utilization Efficiency and Modulates Body Composition and Muscle Function in a Protein Source-Dependent Manner

Iván Benito-Vázquez [1,2], Pablo Méndez-Albiñana [1,3], Aaron Fernández-Quintero [2], María Ines Morán-Valero [2], Francisco Javier Moreno [1], Marina Díez-Municio [2], Nuria Fernández [3], Luis Monge [3], Jose Antonio Uranga-Ocio [4,5] and Javier Blanco-Rivero [3,6,7,*]

[1] Food Science Research Institute CIAL (CSIC-UAM), Nicolás Cabrera 9, 28049 Madrid, Spain
[2] Pharmactive Biotech Products SLU, Faraday, 7, 28049 Madrid, Spain
[3] Department of Physiology, School of Medicine, Autonomous University of Madrid (UAM), 28029 Madrid, Spain
[4] Department of Basic Health Sciences, University Rey Juan Carlos (URJC), 28922 Alcorcón, Spain
[5] High Performance Research Group in Physiopathology and Pharmacology of the Digestive System (NeuGut), University Rey Juan Carlos (URJC), 28922 Alcorcón, Spain
[6] Research Institute of La Paz University Hospital (IdiPAZ), 28040 Madrid, Spain
[7] Center for Biomedical Research Network (CIBER) in Cardiovascular Diseases (Ciber-CV), 28029 Madrid, Spain
* Correspondence: javier.blanco@uam.es

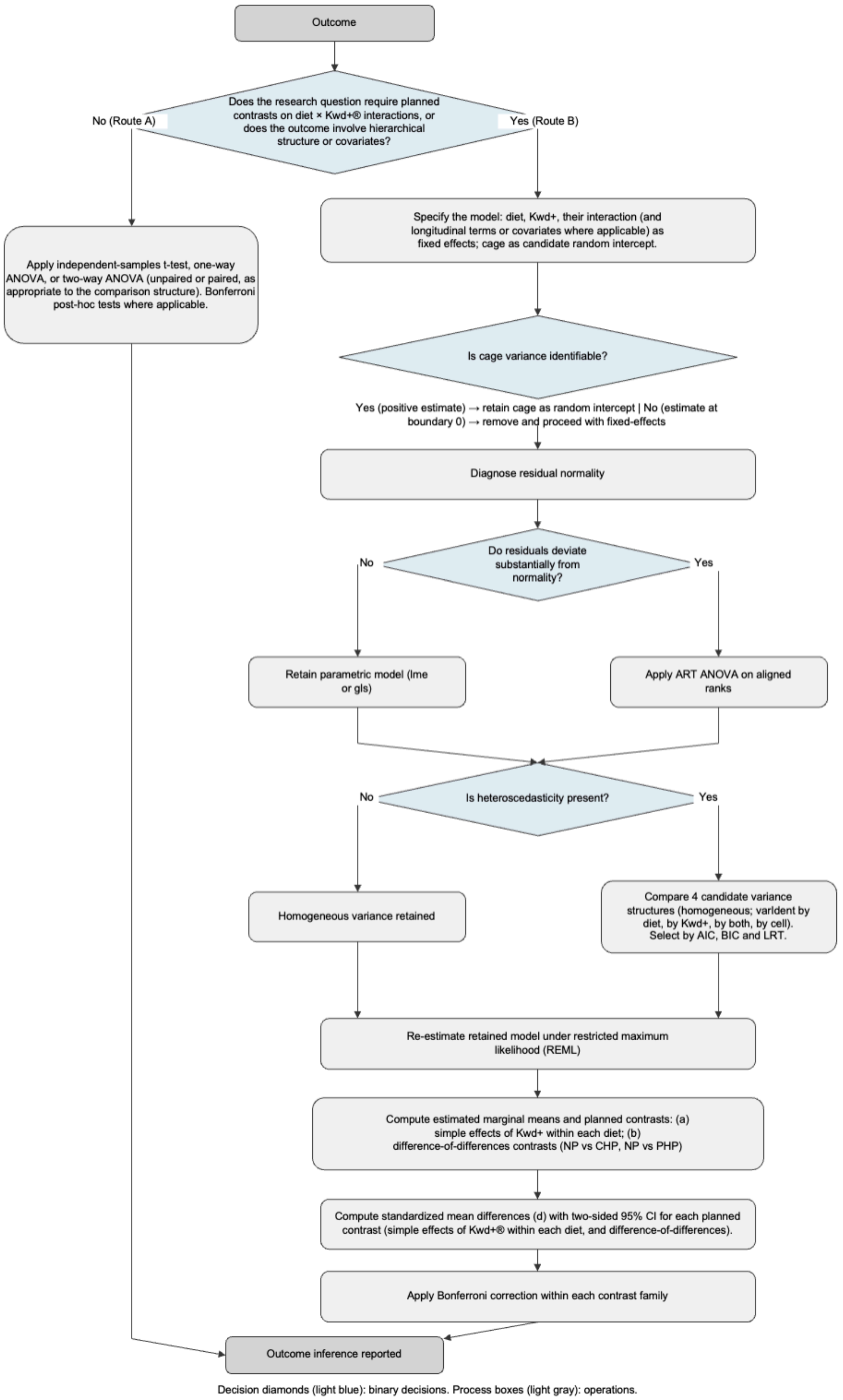

Figure S1. Workflow of the statistical analysis strategy applied for outcome evaluation, including model selection, residual diagnostics, variance structure assessment, planned contrasts, and multiple-comparison correction.

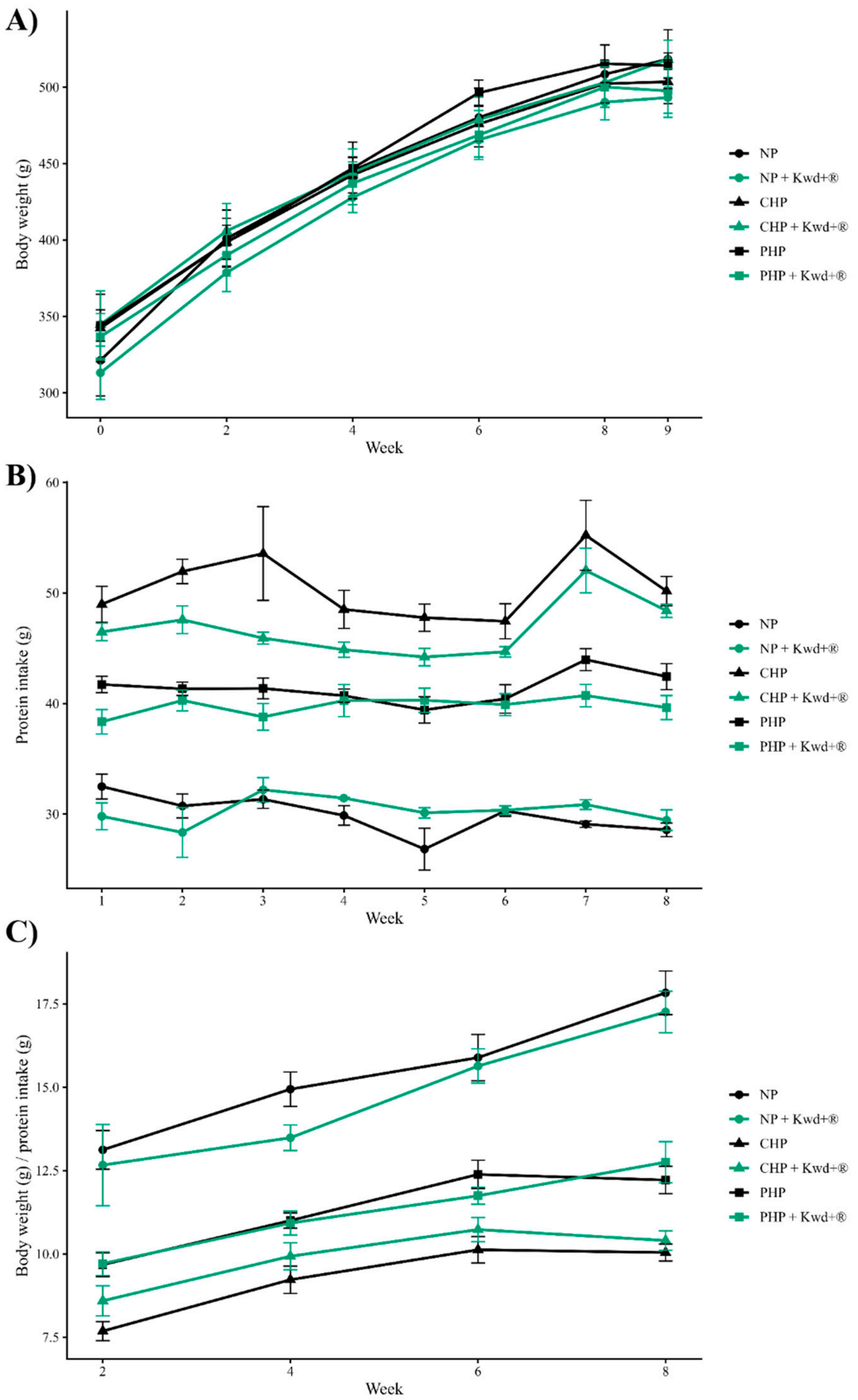

Figure S2. Evolution of **A)** body weight, **B)** protein intake and **C)** weight gain normalized to protein intake during the intervention. Data are presented as mean ± SEM for each dietary group: normoproteic diet (NP), normoproteic diet supplemented with Kwd+® (NP + Kwd+®), casein-based hyperproteic diet (CHP), casein-based hyperproteic diet supplemented with Kwd+® (CHP + Kwd+®), pea-protein hyperproteic diet (PHP), and pea-protein+® hyperproteic

diet supplemented with Kwd+® (PHP + Kwd+®). Circles represent NP diets, triangles represent CHP diets, and squares represent PHP diets.

Table S1. Percentage degradation of casein subunits in stomach chyme prior to gastric emptying under normoproteic and hyperproteic casein diets with Kwd+® supplementation.

Diet	Protein	Degradation (%)
NP	α2-casein	77(15)
	α1+β-casein	90(10)
CHP	α2-casein	86(12)
	α1+β-casein	92(6)

Values are presented as mean (SD). NP, norproteic diet. CHP, high-protein casein diet.

Table S2. Qualitative assessment of pea protein subunit degradation in stomach chyme prior to gastric emptying under hyperproteic diets with or without Kwd+® supplementation.

	Replicate									
Protein	1		2		3		4		5	
	KWD-	KWD+	KWD-	KWD+	KWD-	KWD+	KWD-	KWD+	KWD-*	KWD+
**Convicilin**	+	-	-	-	-	-	+	-	-	-
**Vicilin**	+	-	-	-	-	+	+	+	+	+
**Legumin α (11S)**	+	-	-	-	-	-	+	-	-	-
**Vicilin (7S glob)**	+	-	+	-	+	-	+	-	+	↓
**Vicilin (7S glob)**	-	-	-	-	-	-	+	-	+	-
**Legumin β (11S)**	+	+	+	↓	+	+	+	-	+	+
**LMW 1**	+	↓	+	-	+	↓	+	-	+	-
**LMW 2**	+	↓	+	-	+	+	+	-	+	↓
**LMW 3**	+	-	+	-	+	-	+	-	-	-
**LMW 4**	-	+	-	+	-	+	-	-	+	-

(+) band detected, (-) band not detected, (↓) low band intensity. *A band corresponding to lipooxigenase (LOX) was also detected.

Table S3. Plasma amino acid concentrations (µmol/L) across experimental groups.

Amino acid (µmol/L)	NP	NP-Kwd+®	CHP	CHP-Kwd+®	PHP	PHP-Kwd+®
Alanine	672.06 (63.65)	626.39 (180.50)	576.70 (73.38)	688.99 (123.63)	518.80 (53.63)	567.66 (92.53)
Glutamate	174.97 (34.54)	167.29 (53.05)	155.08 (31.36)	175.40 (45.58)	125.67 (29.68)	114.83 (39.16)
Glutamine	598.24 (48.55)	600.37 (109.54)	570.42 (82.03)	621.60 (113.18)	534.80 (68.66)	539.31 (86.96)
Glycine	226.08 (39.74)	260.50 (71.80)	213.63 (41.74)	231.89 (45.24)	228.38 (42.53)	268.71 (52.29)
Histidine	45.21 (13.25)	51.31 (14.40)	50.15 (10.16)	53.11 (10.01)	48.81 (10.02)	47.95 (11.04)
Threonine	316.50 (56.96)	378.93 (95.45)	321.52 (61.73)	300.09 (90.14)	234.15 (56.96)	306.50 (69.54)
Tyrosine	74.18 (11.04)	73.43 (26.73)	70.55 (11.11)	79.98 (22.22)	61.84 (8.08)	62.57 (11.95)
Valine	204.44 (37.58)	188.69 (43.44)	217.49 (40.45)	227.12 (38.14)	165.22 (16.63)	168.00 (31.37)
Isoleucine	80.95 (18.06)	74.37 (19.91)	79.73 (14.90)	85.19 (16.27)	64.49 (11.39)	68.97 (18.07)
Leucine	157.38 (30.48)	148.13 (37.27)	158.97 (24.36)	167.47 (30.49)	132.49 (14.28)	129.52 (24.41)
Total amino acid	2518.34 (230.53)	2569.42 (551.91)	2414.22 (266.44)	2630.85 (447.69)	2100.69 (133.71)	2274.02 (303.69)

NP: normoproteic casein diet; CHP: high-protein casein diet; PHP: high-protein pea protein diet. Kwd+® indicates supplementation with the actinidin-enriched kiwifruit concentrate. Values are expressed as mean (SD).

Table S4. Twitch parameters recorded in sural triceps from NP and NP-Kwd+® rats.

	**Contraction**					**Relaxation**			
	Maximal contraction	$T_{1/2}$	T_{max}	$Slope_{max}$	$Slope_{25\text{-}75}$	$T_{1/2}$	T_{max}	$Slope_{max}$	$Slope_{25\text{-}75}$
**NP**	146 (14.91)	12.2 (0.2)	24.1 (0.5)	10148(1210)	9287(1093)	15.0(0.5)	54.1(3.6)	- 6448(863)	- 5439(661)
**NP-Kwd+®**	141.4 (16.02)	12.1 (0.4)	23.5 (0.5)	9296 (900)	8747(949)	15.0(1.4)	56.2(3.1)	- 6177(632)	- 4903(533)

T ½: time to half of the contraction/relaxation process (ms); Tmax: time to maximum contraction/relaxation (ms); $Slope_{max}$: maximum slope during the contraction/relaxation process (g/s); $Slope_{25\text{-}75}$: slope between 25% and 75% of the contraction/relaxation process (g/s). Results are expressed as Mean ± SEM. N=10 animals per group.

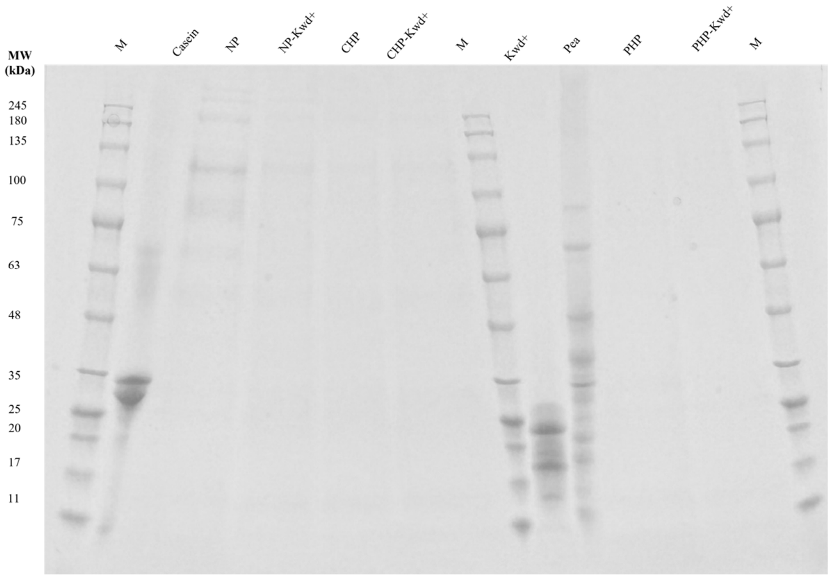

Figure S3. SDS–PAGE profiles of protein hydrolysis patterns in intestinal contents following gastric emptying under the different dietary and Kwd+® supplementation conditions. NP, normoproteic diet; CHP, high-protein casein diet; PHP, high-protein pea diet. M, protein marker.

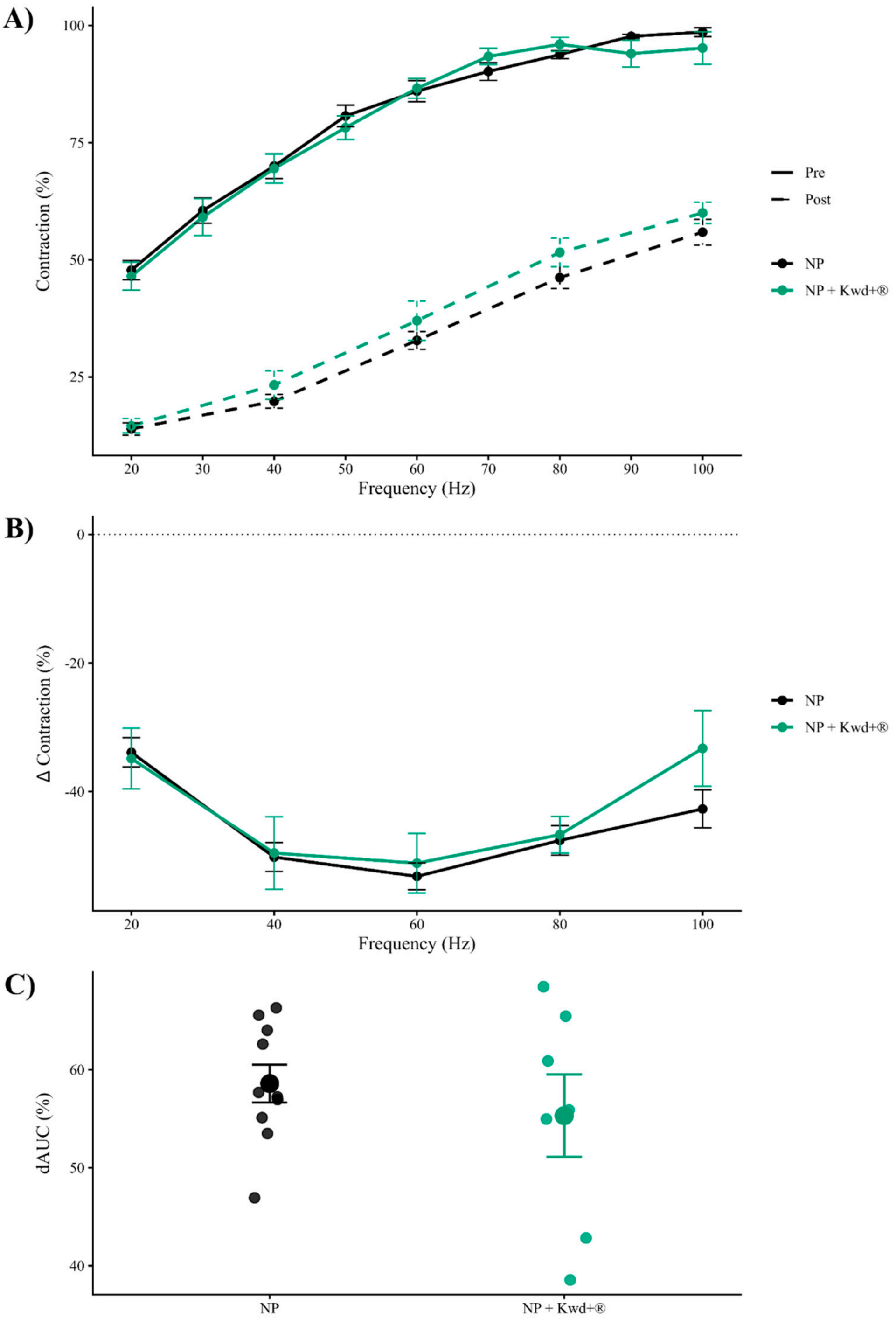

Figure S4. Tetanic force–frequency relationship before and after the fatigue protocol in NP and NP+Kwd+® muscles. **A)** Force–frequency curves obtained before (solid lines) and after (dashed lines) the fatigue protocol across stimulation frequencies (20–100 Hz). Values represent mean ± SEM. **B)** Relative change in contraction force (Δ contraction, %) between pre- and post-fatigue conditions at each stimulation frequency. **C)** Global change in contractile performance calculated as the difference in the area under the force–frequency curve (dAUC, %). Individual points represent single muscles and black symbols indicate mean ± SEM.
